# Reprisal of *Schima superba* to Mn stress and exploration of its defense mechanism through transcriptomic analysis

**DOI:** 10.3389/fpls.2022.1022686

**Published:** 2022-10-06

**Authors:** Fiza Liaquat, Muhammad Farooq Hussain Munis, Samiah Arif, Muhammad Aamir Manzoor, Urooj Haroon, Iftikhar Hussain Shah, Muhammad Ashraf, Hyun Seok Kim, Shengquan Che, Liu Qunlu

**Affiliations:** ^1^ School of Agriculture and Biology, Shanghai Jiao Tong University, Shanghai, China; ^2^ Department of Agriculture, Forestry, and Bioresources, Seoul National University, Seoul, South Korea; ^3^ Research Institute of Agriculture and Life Sciences, Seoul National University, Seoul, South Korea; ^4^ Department of Plant Sciences, Faculty of Biological Sciences, Quaid-i-Azam University, Islamabad, Pakistan; ^5^ National Key Laboratory of Plant Molecular Genetics, CAS Center for Excellence in Molecular Plant Sciences, Institute of Plant Physiology and Ecology, Shanghai Institutes for Biological Sciences, Chinese Academy of Sciences, Shanghai, China; ^6^ Interdisciplinary Program in Agricultural and Forest Meteorology, Seoul National University, Seoul, South Korea; ^7^ National Center for AgroMeteorology, Seoul, South Korea; ^8^ Department of Landscape Architecture, School of Design, Shanghai Jiao Tong University, Shanghai, China

**Keywords:** Manganese, transcriptome, ATP Binding Cassette transporter, hyperccumulater, *Schima superba*

## Abstract

One of the most diverse protein families, ATP-binding cassette (ABC) transporters, play a role in disease resistance, heavy metal tolerance, and food absorption.Differentially expressed genes contribute in the investigation of plant defense mechanisms under varying stress conditions. To elucidate the molecular mechanisms involved in Mn metal stress, we performed a transcriptomic analysis to explore the differential gene expression in *Schima superba* with the comparison of control. A total of 79.84 G clean data was generated and 6558 DEGs were identified in response to Mn metal stress. Differentially expressed genes were found to be involved in defense, signaling pathways, oxidative burst, transcription factors and stress responses. Genes important in metal transport were more expressive in Mn stress than control plants. The investigation of *cis*-acting regions in the ABC family indicated that these genes might be targeted by a large variety of trans-acting elements to control a variety of stress circumstances. Moreover, genes involved in defense responses, the mitogen-activated protein kinase (MAPK) signaling and signal transduction in *S. superba* were highly induced in Mn stress. Twenty ABC transporters were variably expressed on 1st, 5th, and 10th day of Mn treatment, according to the qRT PCR data. Inclusively, our findings provide an indispensable foundation for an advanced understanding of the metal resistance mechanisms. Our study will enrich the sequence information of *S. superba* in a public database and would provide a new understanding of the molecular mechanisms of heavy metal tolerance and detoxification.

## 1 Introduction

Manganese (Mn) is one of the most hazardous heavy metals to contaminate soil and decrease plant yield (Queiroz et al., 2021). Removal of heavy metals from soil is very difficult and it requires complicated efforts. Many physiochemical and biological approaches are being used to decontaminate soils. A low-cost and very effective way to remove metal from contaminated soil is through phytoremediation (Song et al., 2022). The ability of a plant to endure and accumulate heavy metal stress is mostly influenced by heavy metal concentration. species characteristics, the number of rhizosphere microbes, and the concentrations of related metabolites (Barra Caracciolo et al., 2021). Plant species known for Mn hyperaccumulation are typically woody and found in subtropical regions, and they belong to the families Apocynaceae, Celastraceae, Clusiaceae, Myrtaceae, and Proteaceae (Bidwell et al., 2002).

*S. superba* (*Theaceae*) had unusually high Mn levels in the leaves, according to research conducted on a Mn mine land (Yang et al., 2008). This tree is a potential Mn hyperaccumulator, according to Baker and Brooks, who defined metal hyperaccumulation. This tree grows quickly, has a large ecological amplitude, and a lot of biomass, so it has a lot of potential for on-site metal remediation (Liaquat et al., 2021). To acquire tolerance, heavy metal ions absorbed inside the plant were expelled from the cells, reducing heavy metal effectiveness and toxicity. When plants are exposed to heavy metal stress, reactive oxygen species (ROS) are produced, which further obstructs photosynthesis and respiration and seriously harms their membrane systems (Singh et al., 2016). Several proteins and genes that regulate Mn absorption, translocation, and the integration of specific Mn detoxification signal pathways have recently been identified (Tang et al., 2021).

ATP-binding cassette (ABC) transporters are a large protein superfamily found in all living organisms (Hyde, 1990). Most ABC transporters encode membrane-bound proteins that transport a diverse range of molecules across membranes (Dean et al., 2001). ABC transporters are a diverse group of proteins found in all organisms that act as ATP-dependent pumps, ion channels, and channel regulators to mediate cellular trafficking across biological membranes (Holland et al., 2003). ABC transporters exist in several isoforms and are involved in heavy metal detoxification. RNA-Seq data has helped plant scientists to understand the response of plants under different heavy metal stress conditions. However, very few studies have described or clarified the mechanisms of Mn hyper-accumulation (Ai et al., 2018). To understand hyper-accumulation of Mn, studies on uptake, movement and internal detoxification are still not understood very well. Till date, no complete genome of Mn hyper-accumulator is available and it restricts the study of its molecular mechanisms (Pasricha et al., 2021). With the advent of new sequencing technologies like RNA-seq, the availability of these kinds of information can be expected, soon. The aim of this study was to evaluate the differential responses of ABC transporter genes of *S. superba* in response to metal stress.

## 2 Materials and methods

### 2.1 Plant materials

In the glass greenhouse of Shanghai Jiao Tong University, 1-year-old *S. superba* seedlings were used in a pot experiment. Hoagland solution quarter strength was used to water these seedlings. 36 seedlings with similar growth performance (about 35 cm tall) were randomly assigned to two groups: control sample (CK) and the sample treated with 100 mM Mn (WT) for 1, 5 and 10 days. Every group was performed with three biological replicates (CK1, CK2, CK3, WT1, WT2, and WT3). Leaf samples from control and metal-treated plants were taken and rinsed in tap water before being rinsed with ddH2O and dried on sterile absorbent paper.

### 2.2 RNA extraction, library construction and sequencing

In this study, RNA samples were extracted from the control and plants treated with 100 mM Mn for 1, 5, and 10 days using the Agilent 2100 Bioanalyzer (Agilent Technologies, Santa Clara, CA, USA). The samples that had an RNA Integrity Number (RIN) 7 were employed in the study that followed. The TruSeq Stranded mRNA LT Sample Prep Kit (Illumina, San Diego, CA, USA) was used to create the cDNA libraries in accordance with the manufacturer’s recommendations. 125bp/150bp paired end reads were produced from the sequenced libraries using the Illumina HiSeqTM 2500 sequencing technology.

### 2.3 Bioinformatics analysis

#### 2.3.1 RNA Seq quality assessment and genome mapping

Raw data (raw reads) were processed and converted to clean reads using the Trimmomatic software. ^[251]^. FASTQ (also known as fq) files were used to save the raw reads and results, which included sequencing. Trimmomatic was used to separate ploy-N, adopter sequence, and low-quality reads to generate clean reads. The quality of trimmed and untrimmed reads was assessed using FastQC.

#### 2.3.2 Gene-level quantification

Cufflinks was used to calculate the expression level of protein coding gene fragments per kilobase of transcript per million mapped reads (FPKM) (Trapnell et al., 2012) htseq-count was used to calculate the read counts for each protein-coding gene (Pomaznoy et al., 2019).

#### 2.3.3 GO and KEGG enrichment of differentially expressed genes

DESeq was used to assess gene expression differences (Wang et al., 2019). The level of gene expression was calculated using the base mean value. NB calculated the difference multiple and performed the significant difference test on the number of reads (negative binomial distribution test). A threshold of P value (less than 0.05) and fold change (greater than 2) was set for screening differential gene expression. For screening differential gene expression, a P value less than 0.05 and a fold change greater than 2 were used. The gene expression patterns were investigated using hierarchical cluster analysis. To define key biological functions and pathways, all DEGs were mapped to terms in the Kyoto Encyclopedia of Genes and Genomes (www.kegg.jp/kegg/kegg1.html). The hypergeometric distribution was used to perform Gene Ontology enrichment and KEGG pathway enrichment analysis on DEGs. The differential genes between samples were analyzed for MF and BP enrichment using the fisher algorithm, and a directed acyclic graph was created using top 20 GO for the enriched term (Sharma et al., 2019). PCA, hierarchical clustering, and correlation among all samples were carried out to examine the accuracy and consistency of biological copies as well as the variations between stressed and control.

#### 2.3.4 Comparative Phylogenetic and multiple sequence alignment analysis

A comparative study of 30 highly expressed ABC transporters from *S. superba* and 128 proteins from *Arabidopsis thaliana* was carried out using ClustalX software. The Maximum Likelihood approach was used to determine the evolutionary relationship by using online IQ-tree software. All alignments were completed to generate a phylogenetic tree using itol. MEME software (http://meme-suite.org/tools) was used to identify the conserved ABC protein motif (Zhang et al., 2020).

#### 2.3.5 Promoter analysis

Online webtools from the PlantCARE database were used to search for *cis-acting* elements in the promoter sequence. In brief, the promoter sequence was the 1500-bp sequence upstream of the ABC gene family’s ATG start codon. *Cis-acting* elements unrelated to heavy metal stress were removed, and the elements were drawn and visualized using TB tools (Huang et al., 2021).

#### 2.3.6 Real time PCR analysis

The expression of several ABC transporter genes was assessed using q-PCR to validate the transcriptome results. Numerous plant ABC transporters have been found to be involved in the transport of hazardous metals, defending plants from the negative effects of toxic heavy metals (Song et al., 2022). The control gene used was actin. Sangon Biotech manufactured the primers after designing them with the Primer3 software program (Shanghai, China). For each sample, three technical replicates were used. Water devoid of RNase served as the adverse control. The 7500 Real-Time PCR System was used to conduct PCR analysis in three replicates on an optical 96-well plate (Applied Biosystems). The PCR mixture was made using the Huang et al., 2022 technique. Thermal cycling started with denaturation at 95°C for 1 minute, then 40 cycles of 95°C for 15 seconds, 57°C for 15 seconds, and 72°C for 15 seconds (45 s). The list of qPCR primers found in **Table 1**.

**Table 1:** Primer sequences of selected genes for q-PCR.

No.	Gene ID	Gene Name	Primers
1	F01_transcript_122	*SsABCc2*	F CGGGACTGTTGCCTATGTTT
			R TGCTTCTGACCACCACTGAG
2	F01_transcript_167	*SsABCc3*	F CTGCAAGCTTGGGAGGATAG
			R TTGGAGGATCCTGAAAGTGG
3	F01_transcript_262	*SsABCc6*	F TGTGAGTGGCTATGCCTCAG
			R AAACCGGTGAAAGCAATGTC
4	F01_transcript_57784	*SsABCc9*	F GGGCTTGAGGTTGTCATGTT
			R TTTTGTGCCCATCAATACGA
5	F01_transcript_65017	*SsABCc11*	F TGAAGAAGGGCAAGGAGAAA
			R TTCCGCTGGCAGAAGAGTAT
6	F01_transcript_70595	*SsABCc13*	F CAGGTTAGGTATCGCCCAAA
			R TTGAGGAATGATCCCAAAGC
7	F01_transcript_71372	*SsABCc15*	F GTTTCGAGCACCAATGTCCT
			R TTTCTGCATCCACAAGCAAG
8	F01_transcript_84527	*SsABCc17*	F CTTGTTTCGCCTGGTAGAGC
			R CCTCAACAACCTCTCGAAGC
9	F01_transcript_86030	*SsABCc19*	F ATTGCAGGGTTGGCAGTAAC
			R TCAATACGGAAGGGAGATGC
10	F01_transcript_62452	*SsABCc73*	F AGCGAAGCCCCTGAAATAAT
			R GCTCTACCAGGCGAAACAAG
11	F01_transcript_39199	*SsABCd1*	F GACTCTCCGAAGCTCCTCCT
			R CAACACTGCCCCCTGTAACT
12	F01_transcript_81781	*SsABCf1*	F TGTGGGTGGTCGTGAACTTA
			R GCTGCGTTCTTTCAATGTCA
13	F01_transcript_53446	*SsABCg1*	F TCCTCTGAGCGAGACCTTGT
			R CCCATTAGTGCCGTGAAACT
14	F01_transcript_56703	*SsABCg2*	F GTGGTGGGGAGCATAAGAGA
			R GTTTCACCGCCCGATAGTAA
15	F01_transcript_8233	*SsABCg5*	F GTGGGATCAGTGGAGGAGAA
			R TTTGCCTCCAGAAAGCAAGT

### 2.4 Data analysis

The results were presented as mean standard deviation (SD) and analyzed using one-way ANOVA (ANOVA, *P <* 0.05).

## 3 Results

### 3.1 Illumina sequencing and quality control

To elucidate the molecular responses to Mn stress in *S. superba*, we prepared 6 libraries from Mn treated and control samples of the transcriptomic sequencing and 124.45G of clean data was obtained. The Q30 base distribution was 94.37~94.99%, and the average GC content was 46.48% (**Table 2**).

**Table 2:** Sample sequencing data evaluation statistics table.

Treatment time	Replicates	Read number	Base number	GC content	%≥Q30
1 day	CK1	23,876,897	7,114,912,142	47.04	94.64
5 day	CK5	25,589,463	7,628,579,546	45.97	94.45
10 day	CK10	20,404,241	6,079,274,534	45.74	94.90
1 day	T1	23,321,945	6,955,239,038	47.18	94.88
5 day	T5	20,469,957	6,102,017,436	45.80	94.85
10 day	T10	22,696,113	6,772,220,786	46.16	94.61

### 3.2 Alignment and In silico analysis

**Figure 1:**
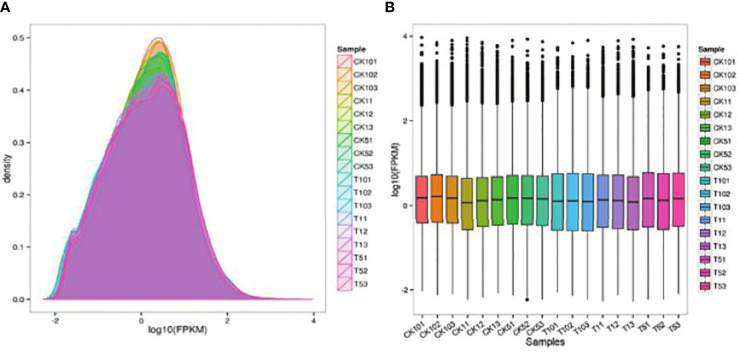
**(A)** The FPKM density distribution reflects each sample’s protein-coding gene expression pattern. **(B)** FPKM box diagram for each sample.

This project used non-redundant transcripts, measured in three generations as references for sequence alignment and subsequent analysis. STAR was performed to compare Clean Reads with transcripts to get position on the transcript (**Table 3**). The protein-coding gene expression profile of each sample is represented by the FPKM density distribution (**Figure 1A**). The FPKM distribution of genes in each sample was represented by an FPKM density map for all sample genes. Each sample expression value (FPKM) was separated into various intervals due to the variations in the samples’ gene expression values and number of expressed genes. Stacked histograms were made when the number of genes expressed in various expression interval samples was established (**Figure 1B**).

**Table 3:** Comparison results between second-generation sequencing data and non-redundant transcripts measured in third-generation.

Sample	Total Reads	Uniquely mapped reads %	% of reads mapped to multiple loci	% of reads mapped to many loci
CK1	29,895,001	29.02%	51.03%	6.81%
CK5	31,768,485	28.64%	49.46%	6.91%
CK10	21,654,490	27.33%	49.66%	7.68%
T1	23,328,452	31.61%	44.63%	7.39%
T5	20,475,210	34.45%	45.01%	4.28%
T10	26,325,333	35.31%	44.81%	3.04%

### 3.3 Functional annotation and enrichment analysis of differentially expressed transcripts

The functional annotation of the database was performed on the differentially expressed transcripts. The statistics of the number of transcripts annotated in each differentially expressed transcript set shown in (**Table 4**).

**Table 4:** Statistics of the number of annotated differentially expressed transcripts.

#DEG-Set	Annotated	Nr	GO	COG	KEGG	KOG	Pfam	Swiss-prot	Egg-Nog
CK 1st day vs T 1st day	37,850	37760	30458	17029	16161	24268	32348	28398	37272
CK 5th day vs T 5th day	42,757	42655	34333	19179	18186	27404	36333	31983	42108
CK 5th day vs T 5th day	46,179	46076	37119	20583	19605	29516	39074	34593	45453

### 3.4 Statistics and profiling of differential gene expression

Based on the levels of protein-coding gene expression in various samples, differential screening was done. There were three distinct groups. The total number of DEGs was discovered using the FC>2 and P < 0.05 thresholds to be (CK1 vs T1) 47,292, (CK5 vs T5) 38658, and (CK10 vs T10) 43,705, respectively. During the (CK1 vs T1) comparison, 18,602 genes were upregulated while 20,056 were downregulated. In (CK5 vs T5), there were 20,270 upregulated genes and 20,056 downregulated genes. 22,456 genes were increased in CK10 compared to T10, whereas 24,836 genes were downregulated. (**Table 5**).

**Table 5:** Statistics of the number of differentially expressed transcripts.

#DEG-Set	All-DEG	Up-regulated	Down-regulated
CK 1st day_vs_T 1s t day	38,658	18,602	20,056
CK 5th day vs T 5th day	43,705	20,270	23,435
"CK 10th day vs T 10th day"	47,292	22,456	24,836

**Figure 2:**
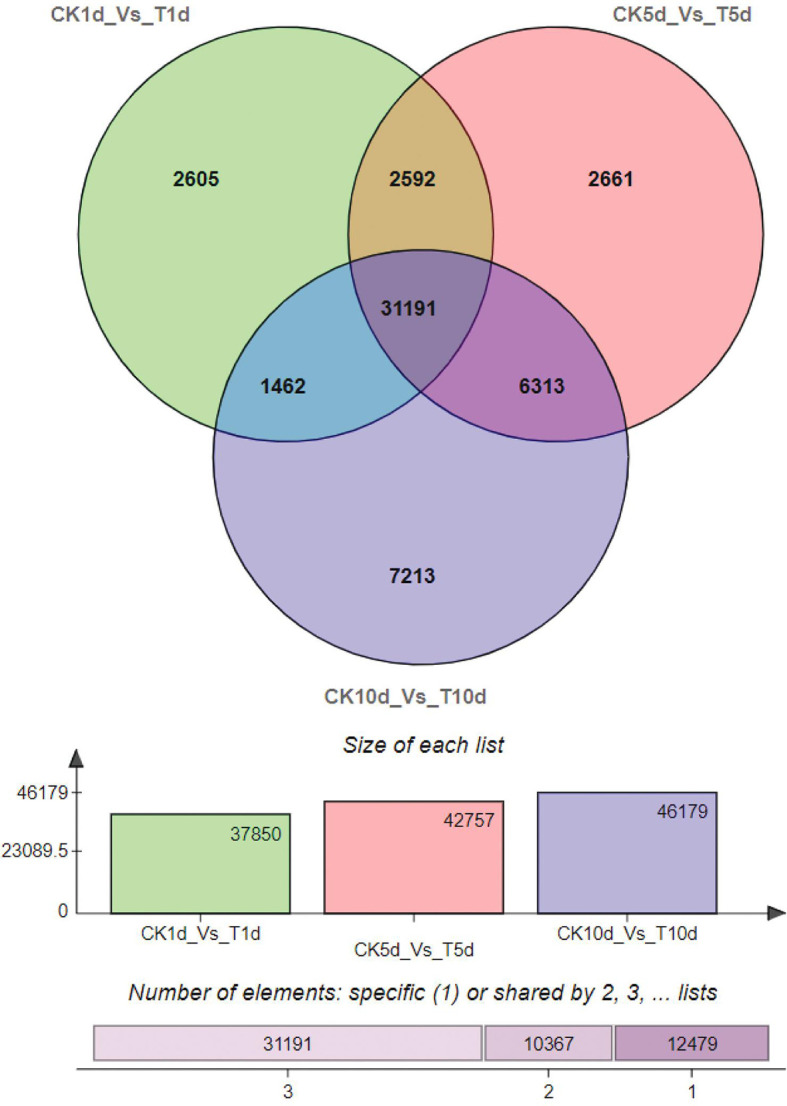
Gene expression profile between the Mn treatment and control.

The three comparisons resulted in a total of 56,145 genes being differentially regulated, of which 37, 850, 42,757, and 46,179 DEGs were found in CK1d-Vs-T1d, CK5d-Vs-T5d and CK10d-Vs-T10d respectively is shown in the (**Figure 2**) below:

### 3.5 Gene ontology and KEGG analysis of DEGs

**Figure 3:**
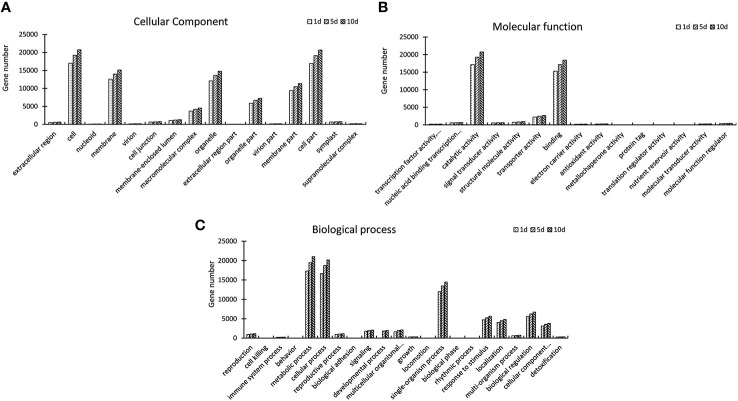
Gene Ontology (GO) classification of *S. superba* unigenes. **(A)** Cellular components; **(B)** Molecular function; **(C)** Biological process.

**Figure 4:**
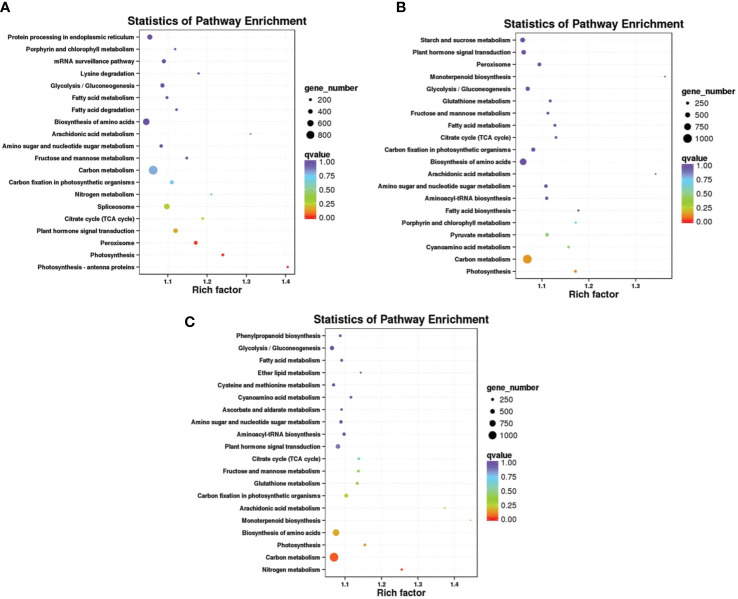
Differentially expressed transcripts KEGG pathways enrichment scatter plot.

GO assignments were used to classify the functions of DEGs, and the result of significantly enriched GO terms (*P*_adj_-value < 0.01). We performed a GO enrichment analysis of the DEGs from three comparisons. The most abundant GO cellular process terms after Mn treatment were in the cell, call part, membrane or in the organelle. Analysis molecular function showed that these target genes were enriched in binding and responding to catalytic activity. In biological process metabolic, cellular, and single- organism process was enriched after Mn treatment. The number of DEGs in the three functional items increased significantly on days 1, 5, and 10, with a visual induction from day 1 to 10. The results of GO enrichment for DEGs at each time point indicated that these DEGs were actively expressed after Mn stress (**Figure 3**). The Kyoto Encyclopedia of Genes was classified based on a pairwise comparison of CK1 VS T1, CK5 VS T5, and CK10 VS T10. Based on these findings, DEGs involved in carbon metabolism, amino acid biosynthesis, and protein processing in the endoplasmic reticulum were found to be highly enriched (**Figure 4**).

### 3.6 Evaluation of sample variation

**Figure 5:**
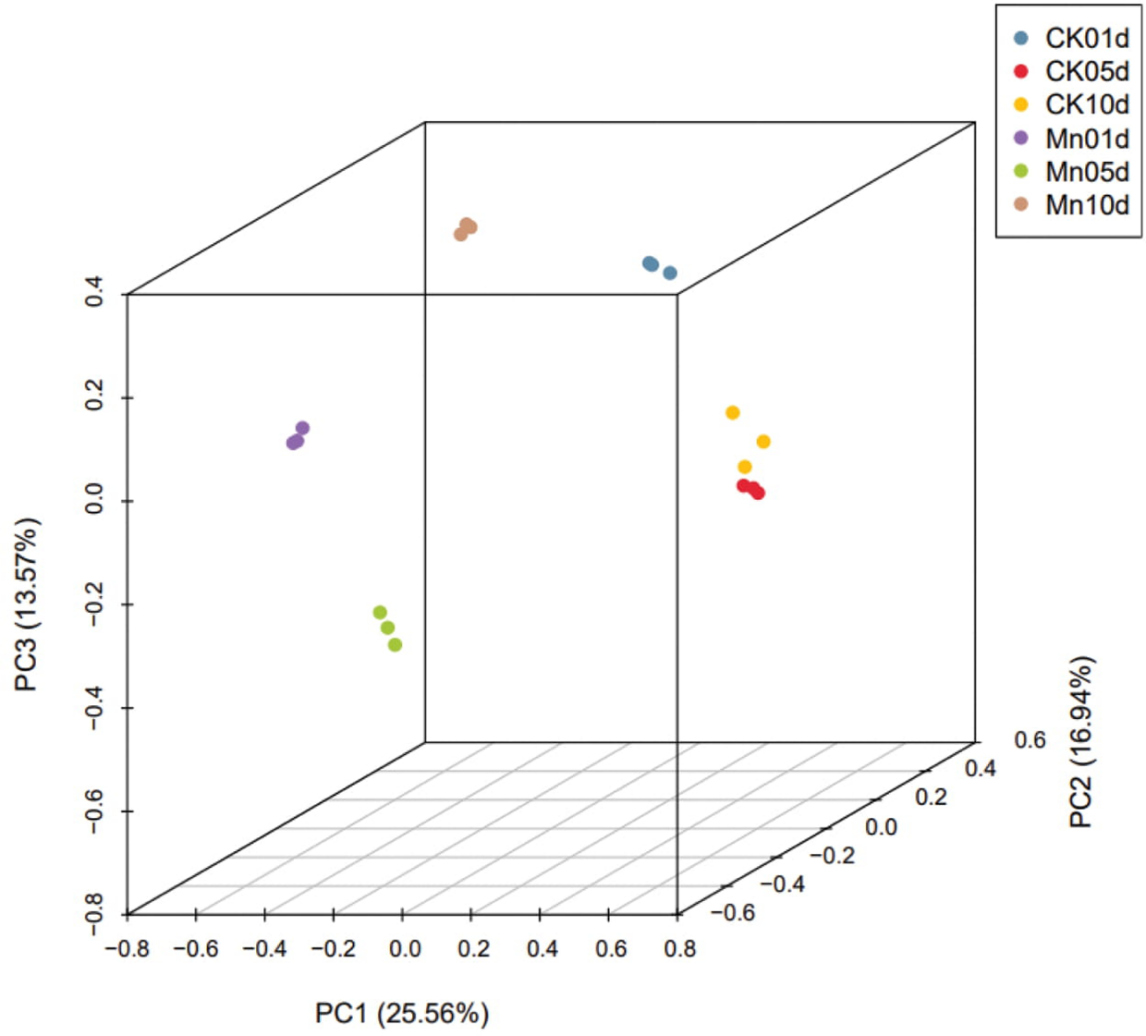
PCA expressing DEG variance in all four data sets.

PCA revealed discrete behavior in control and Mn-stressed plants. All three PC contributed 56 percent of the total variance. PC1 had a variance of 25.56 percent, while PC2 and PC3 had variances of 16.94 percent and 13.57 percent, respectively (**Figure 5**).

### 3.7 Classification of ABC genes in *S. superba*

**Figure 6:**
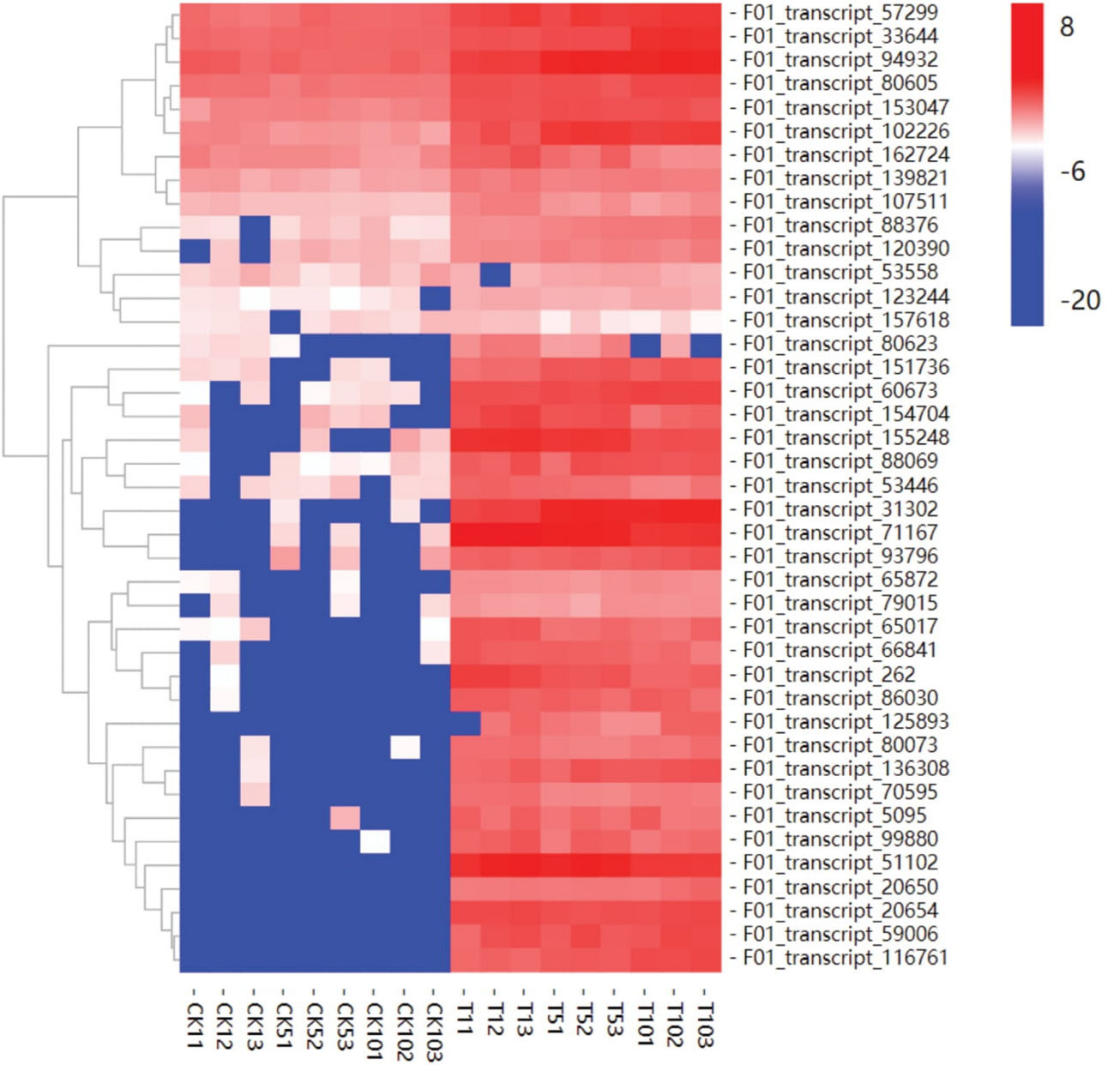
Heat map of the expression level correlation of genes involved in ABC transporters.

From the transcriptome database of *S. superba*, 30 genes from the ABC family were found after incomplete or redundant sequences were removed. 129 members of the Arabidopsis ABC family and the *S. superba* ABC family were used to build a phylogenetic tree, which was then given the original IDs of these genes. (**Figure 6**). The findings show that ABC gene members are divided into five clusters.

**Figure 7:**
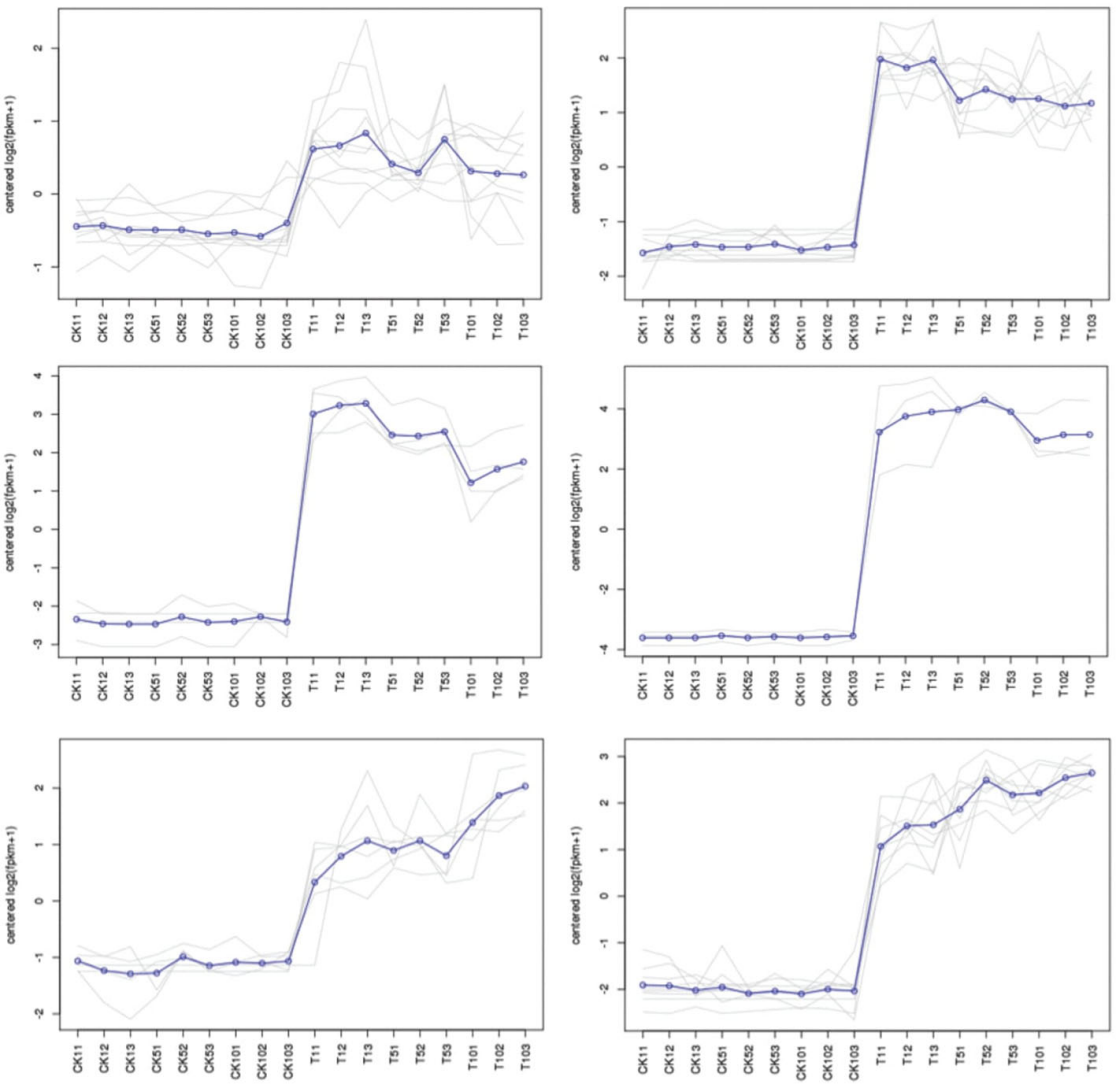
Analysis of the DEGs’ expression trend patterns.

At the beginning of the first day, the expression pattern of Cluster 1 demonstrated an up-regulation trend, which was followed by stable expression in the stage after that. It’s interesting to see that cluster 6 showed an uptick in expression throughout the stage, peaking at day 10. After 1 day, 5 days, and 10 days, clusters 2, 3, and 4 displayed an upregulation tendency, followed by a minor decline. The results suggest that Mn stress can quickly trigger the genes in cluster 1, but clusters 2, 3, and 4 displayed a time-dependent trend in the process (**Figure 7**).

The base determines the value of ratios. Each cluster’s gene expression trend was analyzed using 2 logarithms. The ratio is determined for each gene by dividing the FPKM of the gene in the sample by the FPKM of the gene in the control.

### 3.8 Physiochemical properties of ABC genes

It was found that F01_transcript_167 was the largest identified protein with a range of 1534 amino acids, whereas the smallest one was F01_transcript_52963 with 58 amino acids. The relative molecular weight of these ABC genes varied according to protein size and ranged from 6,137.11 kDa to 170,475.56 kDa, with an average molecular weight around 77,239.44 kDa. It was estimated that the isoelectric point (pI) had a range from 5.39 to 10.37 and the average isoelectric point was 7.48 (**Table 6**).

**Table 6:** Physiochemical properties of ABC transporters.

Gene Id	Amino acid	Molecular weight	Theoretical pI
F01_transcript_99	1193	132880.43	6.15
F01_transcript_122	1477	165097.73	7.86
F01_transcript_167	1534	170475.56	8.16
F01_transcript_220	1521	168710.71	7.65
F01_transcript_262	1510	169161.94	8.09
F01_transcript_26721	541	60562.35	5.65
F01_transcript_39199	323	35158.61	7.02
F01_transcript_52963	58	6137.11	7.98
F01_transcript_53446	258	27961.67	5.71
F01_transcript_55406	79	8842.3	6.04
F01_transcript_55652	249	27871.01	6.60
F01_transcript_56703	703	77694.42	9.23
F01_transcript_57784	842	95163.46	8.73
F01_transcript_60257	300	32993.37	8.07
F01_transcript_62452	527	58960.39	6.20
F01_transcript_63261	335	36996.86	10.37
F01_transcript_65017	849	95556.08	8.55
F01_transcript_65208	136	15138.8	8.00
F01_transcript_71347	847	95243.66	9.11
F01_transcript_71372	925	102557.98	6.68
F01_transcript_73753	227	25544.12	9.86
F01_transcript_76958	648	73634.65	7.96
F01_transcript_81781	515	58204.12	5.39
F01_transcript_82646	1284	143871.86	8.64
F01_transcript_8233	713	79390.58	8.45
F01_transcript_84527	664	74358.91	5.64
F01_transcript_85194	549	60920.56	6.24
F01_transcript_86030	702	78101.28	5.63

### 3.9 Hierarchical cluster analysis and expression pattern of transport-related genes

**Figure 8:**
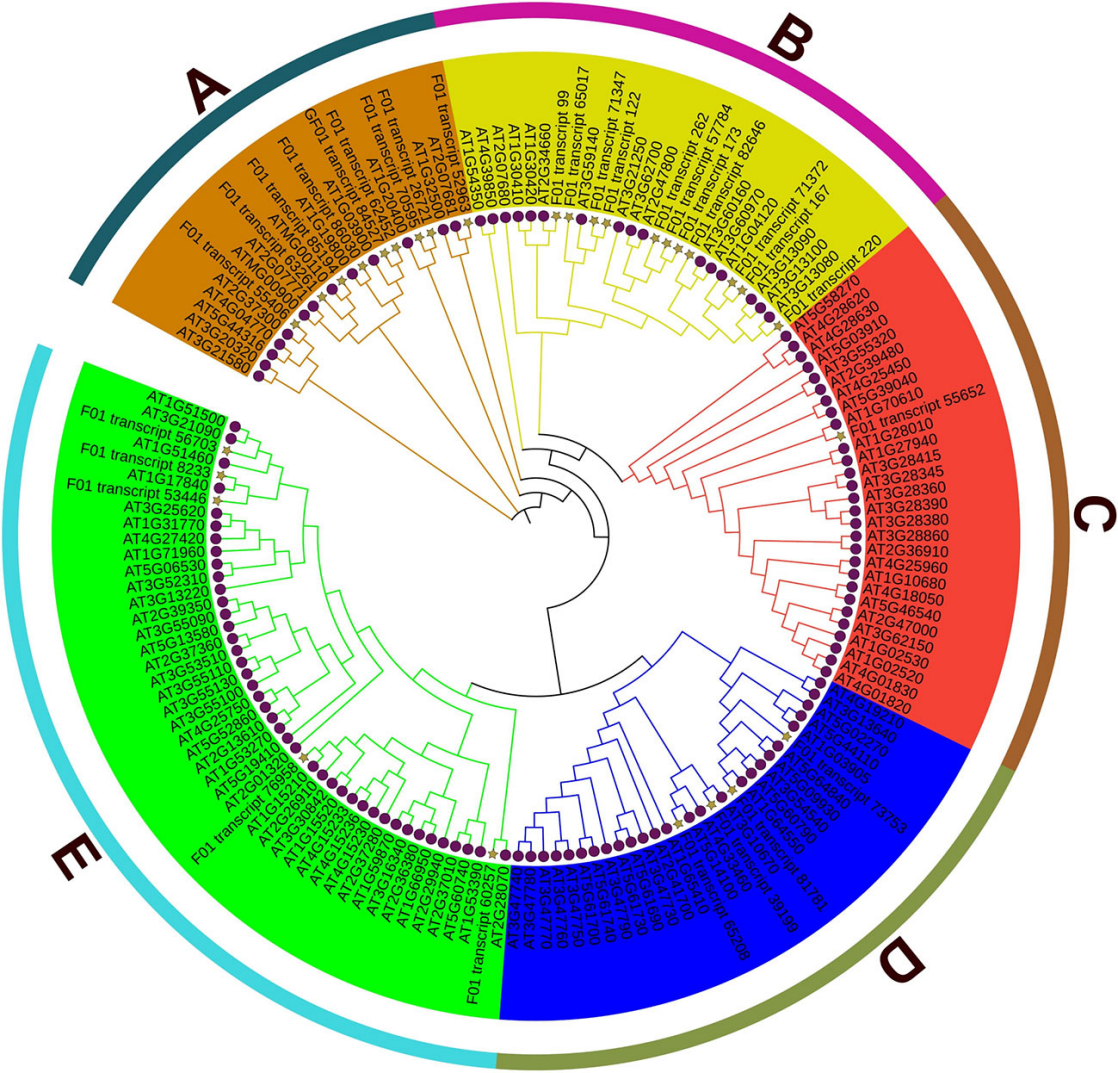
Phylogenetic tree of *S. superba* and *Arabidopsis* genes.

ABC transporters are essential for plant resistance to heavy metal stress. ABC transporters were found to be highly expressed in response to Mn treatment after 1, 5, and 10 days. ABC transporter genes may be involved in Mn detoxification and play important roles in transporting excess Mn from the root to the leaf in plants, which may be another feature of *S. superba’s* hyperaccumulation capacity. When compared to the control, transcripts-51102 and 71167 were significantly upregulated after 1, 5, and 10 days of Mn treatment, and the expression pattern was consistent. In our study, most uni-genes encoding *S. superba* ABC transporters were upregulated throughout the Mn treatment response stage (**Figure 8**). These findings were consistent with ATP binding and intracellular protein transport being enriched functions.

### 3.10 Promoter *Cis-*acting analysis

TFs (transcriptional factors) use binding of *cis*-regulatory elements in the promoters of target genes to regulate them both regionally and functionally (Shibata et al., 2016). The binding specificity of the TFs is determined by the *cis*-regulatory element in the promoter region and plays a key role in transcription regulation. *Cis*-regulatory elements of ABC transporters in *S. superba* were identified to be involved in stress response. The CAAT-Box and TATA-box motifs were discovered in most *ABC* genes, and the number of them was higher in the promoters of transcript-52693, transcript-55652, transcript-82646 and trancript-84527 as compared to other ABC genes. This suggested that TATA-Box and CAAT-Box motifs perform a significant role in the stress response. These *cis*-elements had a role in ABA responsiveness as well as promoter and enhancer regions. Moreover. Light responsiveness cis-acting regulatory elements (Box 4) comprise only 1% of the total *ABC* members. Stress-responsive element was determined ARE (3%) which is associated to light stress (**Figure S1**). These findings suggested that members of ABC gene family could improve metal stress response.

### 3.11 Gene ontology annotation of ABC genes

GO enrichment analysis was used to predict subcellular localization, molecular function, and biological process (**Figure S2**). The predicted distribution scores of ABC transporter proteins in subcellular localization analysis were as follows: 22% in the plasma membrane, 2% in the cytoplasm, and 2% in the chloroplast. The collective scores of ABC transporters during biological process were transport and homeostatic process 8.36% and 7, 95% involved in the response of stress.

Gene ontology depicted the distribution of each ABC gene in the plant, with a brown column representing the cellular compartment. The biological process in which the ABC family participates is shown in red, and the molecular function and subcellular localization are shown in purple and blue.

### 3.12 qRT-PCR analysis of DEGs

**Figure 9:**
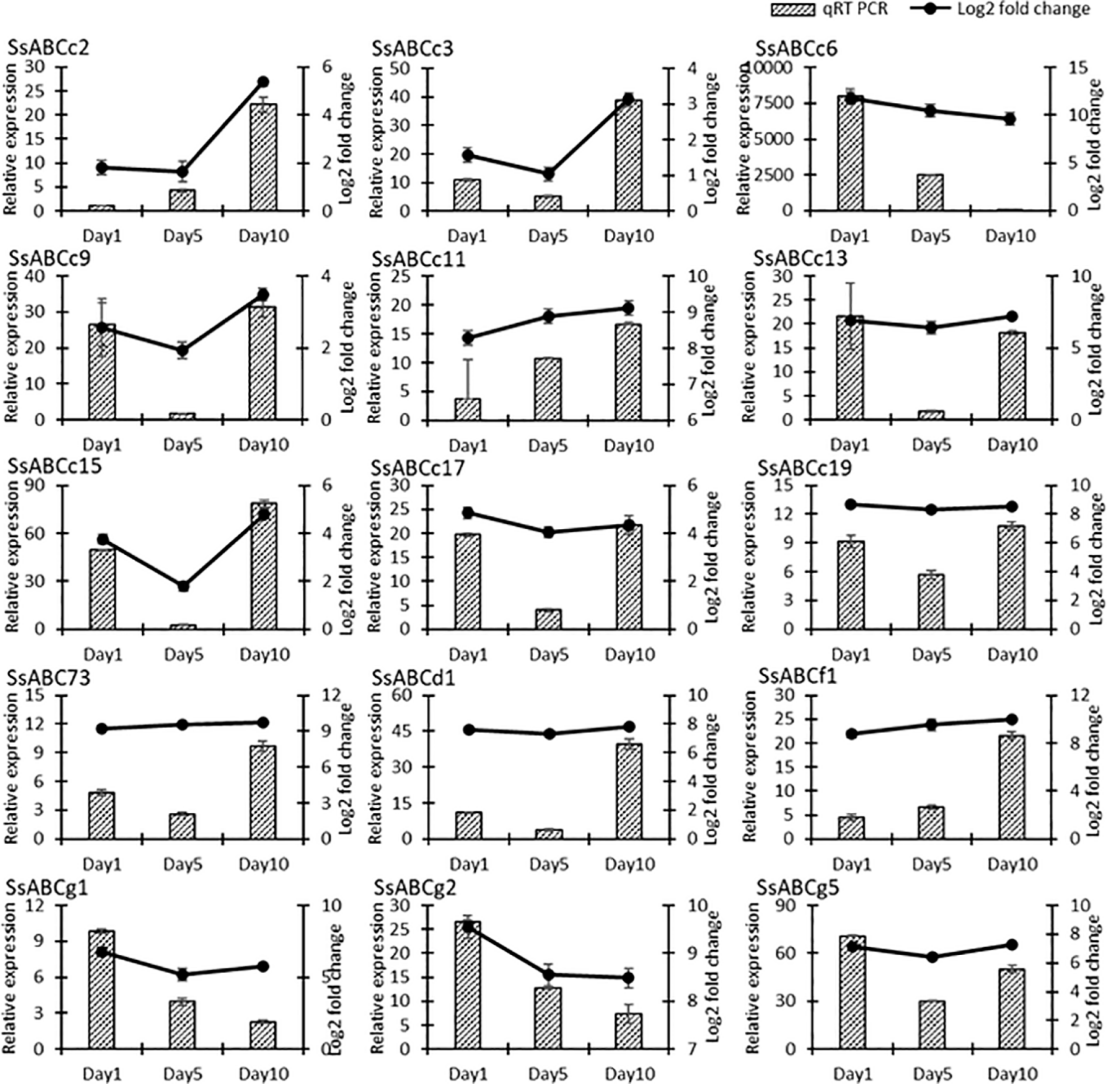
Relative expression of ABC genes in *S. superba* under 100 mM Mn treatment.

To further investigate the funcion of ATP-binding cassette (ABC) transporters and which of the identified transporters could be potentially involved in the regulation of Mn in *S. superba*, 11 ABC genes transporter genes were selected for qRT PCR. It was observed that all selected genes showed various expression levels after 1, 5 and 10 day of Mn treatment, as compared to control. In general, *SsABCc2, SsABCc3, SsABCc9, SsABCc11, SsABCd1 and SsABCf1* had the highest expression in the Mn treated group (WT) on day10, whereas *SsABCc6, SsABCc13, SsABCg1, SsABCg2* and *SsABCg5* had the highest expression on day 1 in the Mn treated group (WT) (**Figures 9**). The alteration patterns of these genes were consistent with that of transcriptome analysis, indicating that the DEGs identified by comparative transcriptome analysis were reliable.

## 4 Discussion

Transcriptomic analysis helps to understand the behavior of any plant under stress conditions. To explore metal stress mechanism and tolerance level, *S. superba* was stressed and examined under different concentrations of Mn. Physiological, proteomics and functional genomics studies have been reported to study metal stress resistance of plants (Hossain and Komatsu, 2013). In this study, the differential expression of transporter genes in *S. superba*, in response to Mn stress has been studied for the first time. In this study, 124.45 G clean data were obtained by doing sequence analysis of *S. superba* under Mn stress. The sequence accuracy was determined through Q30 base distribution that showed 94.37~94.99%, with average GC content 46.48%. Our previous study, using PacBio sequencing revealed almost the same sequence pattern (Liaquat et al., 2021).

In this study, gene function and expression analysis was further performed to see their role in the process of Mn tolerance in *S. superba*. To compare gene expression differences between various samples, protein coding gene expression pattern was analyzed, using FPKM density distribution. Our results indicated that each sample expression value (FPKM) was distributed into different intervals which agrees with previous studies (Filloux et al., 2014). The Pearson Correlation Coefficient was used to evaluate linear relation among gene expression level of the sequencing samples (Koch et al., 2018). Our results suggested maximum difference of gene expression level between the considered samples. Previous studies also showed maximum differences among two samples (Arora et al., 2020). It also indicates that samples belong to same cluster possess similar biological functions (Bushel at al., 2018). Many transcriptomic studies revealed expression patterns of specific DEGs and various profiles of metal stress tolerance (Fan et al., 2021). These studies also indicated that selective induction and rapid activation of metal tolerance pathways might be the primary reason for metal resistance in specific plants. Similarly, our results revealed DEG expression where some genes were upregulated while others were down regulated (**Tables 5**). Differential gene expression may be a result of variations in genotype, interaction between genes under various pathways and environmental conditions (Barbey et al., 2020). We utilized assembled transcriptome for further applications of *S. superba.* Recent studies have reported some metal tolerance genes that encode transmembrane proteins by combining RNA seq, physiological data and SNP analysis (Zhou et al., 2022).

The three GO terms analyzed, such as biological process, molecular function, and subcellular components, provide an important basic classes (Shah et al., 2022). GO is a standardized functional classification analysis that present information of different genes properties and their products in any organism (Balakrishnan et al., 2013). The Go ontology provide basic function of genes based on their predicted function (Manzoor et al., 2021). The assimilation of GO and KEGG pathways presented a broad understanding of various responses to salt stress in various tissues of *S. superba.* Our GO enrichment analysis after 1, 5 and 10 days revealed almost same pattern for biological, cellular, and molecular functions. It indicates that different genes of *S. superba* cooperate with each other to fulfill their biological functions. Our GO analysis indicates that DEGs were involved in metabolic processes, macro and supra molecular complex, transporter activities, stimulus responses which suggest that DEG in this cellular complex would be involved in metal tolerance in plants (Raza et al., 2022). Furthermore, DNA binding transcription factor activity, extracellular region, detoxification process and biological regulation have been reported to exhibit metal tolerance in different plants (Li et al., 2022; Sabir et al., 2022). The DEGs involved in metal ion binding process are likely to promote metal tolerance of plants *via* regulation of downstream target gene’s expression. We also determined that uni-genes were mapped to Mn stress tolerance, based on KEGG pathway. Among them, maximum genes were associated with the biosynthesis of amino acids, carbon metabolism, and plant hormone signal transduction indicating that these pathways may facilitate plants to cope with severe environmental conditions such as drought tolerance, metal stress and saline stress (Dos Santos et al., 2022).

Across a variety of biological membranes, ABC proteins (C-subfamily) act as powerful transporters to enable chemical exchange. To better understand cellular processes, drug development, and tissue expression in humans, ABCC transporters have undergone significant research on substrate selection, tissue expression, and transport kinetics (Niu et al., 2021). ABCC transporters were first discovered in plants as GS conjugate vacuolar pumps, and they were thought to play a role in detoxification, PTE sequestration, chlorophyll catabolite transport, and ion channel modulation. *SsABCc19, SsABCc15*, and *SsABCc17* are three well-studied transporter genes (Lee et al., 2005). ABCC transporters are significant detoxifiers that sequester metal-chelators into plant vacuoles. According to one research, ABCC proteins are involved in PTE hypertolerance and hyperaccumulation (Fasani et al., 2022). Understanding the involvement of ABCC proteins in *S. superba* is crucial for understanding hyperaccumulation of *S. superba*.

The composition and diversification of the ABCC subfamily in *S. superba* were determined using bioinformatics methods, and their expression profiles were assessed for potential role in Mn tolerance and accumulation. Variable expression of ABC transporters has been reported in other crops (Lopez-Ortiz et al., 2019). Plant ABC transporters are crucial membrane proteins that transport and distribute a variety of metabolites and xenobiotics, including heavy metals (e.g., Zn, Mn and Cd). They play a variety of roles in stress, growth, and plant development responses (Gill et al., 2021). They are important in seed germination, stomatal movement, lateral root formation, and other stress responses in plant (Xu et al., 2022). ABCC-type transporters have recently been discovered to be important apo-phytochelatin and phytochelatin-heavy metal (oid) complex transporters (Park et al., 2012). ABCCs have been linked to detoxification and the potential sequestration of harmful metals in plants. In contrast, the *AtABCC3* gene encoded a PC-Cd complex transporter. *AtABCC1* and *AtABCC2* genes have been connected to the phytochelatin vascular sequestration (PC)-Hg (II) and PC-Cd (II), respectively (Nosek et al., 2020). A crucial component of glutathione-mediated detoxification is played by wheat ABCC protein (TaABCC13), while rice ABCC protein (OsABCC1) decreases the quantity of arsenic in grains by securing it in vacuoles (Feng et al., 2020). According to these findings, ABCC members play an important role in determining how hazardous metals are transported and detoxified (Li et al., 2022). *Arabidopsis thaliana* ABCG genes were found to be more resistant to very hazardous heavy metals (Wang et al., 2019).

## Data availability statement

The datasets presented in this study can be found in online repositories. The names of the repository/repositories and accession number(s) can be found in the article/**Supplementary Material**.

## Author contributions

LQ designed the experiments. FL executed the experiments and wrote the manuscript. MFM, MAM analyzed the results and formatted the manuscript. SA, MA and SC supervised manuscript write up. MAM and IH data compilation. UH collected and HK supervised the research work. SC facilitated the team with his lab facilities. All authors read and approved the final manuscript.

## Funding

This research is financially supported by Shanghai Sciences and Technology Commission Project No: 18DZ2283500.

## Conflict of interest

The authors declare that the research was conducted in the absence of any commercial or financial relationships that could be construed as a potential conflict of interest.

## Publisher’s note

All claims expressed in this article are solely those of the authors and do not necessarily represent those of their affiliated organizations, or those of the publisher, the editors and the reviewers. Any product that may be evaluated in this article, or claim that may be made by its manufacturer, is not guaranteed or endorsed by the publisher.
