# Supplementary figures and images for: Reprisal of Schima superba to Mn stress and exploration of its defense mechanism through transcriptomic analysis
Source: Front Plant Sci. 2022 Oct 6;13:1022686. doi: 10.3389/fpls.2022.1022686 (PMC9615920; doi:10.3389/fpls.2022.1022686)

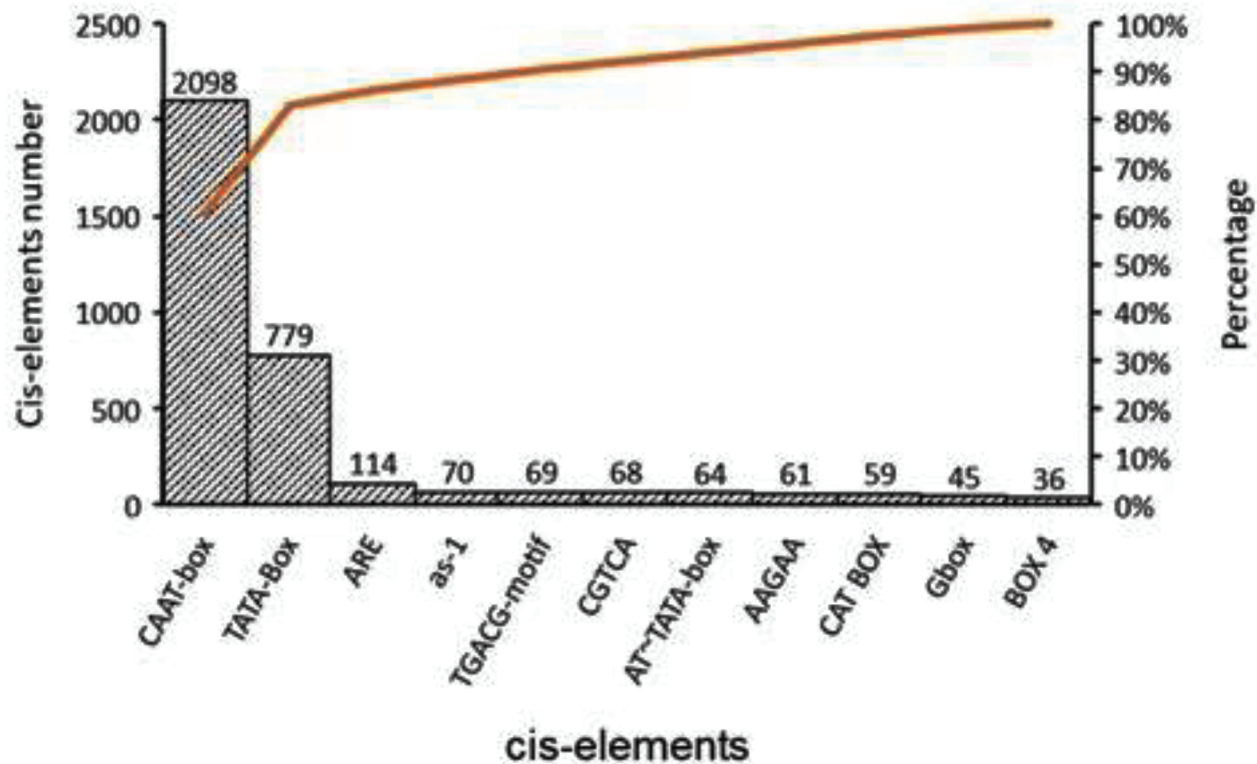

Supplement: Supplementary file 1 [file DataSheet_1.pdf]

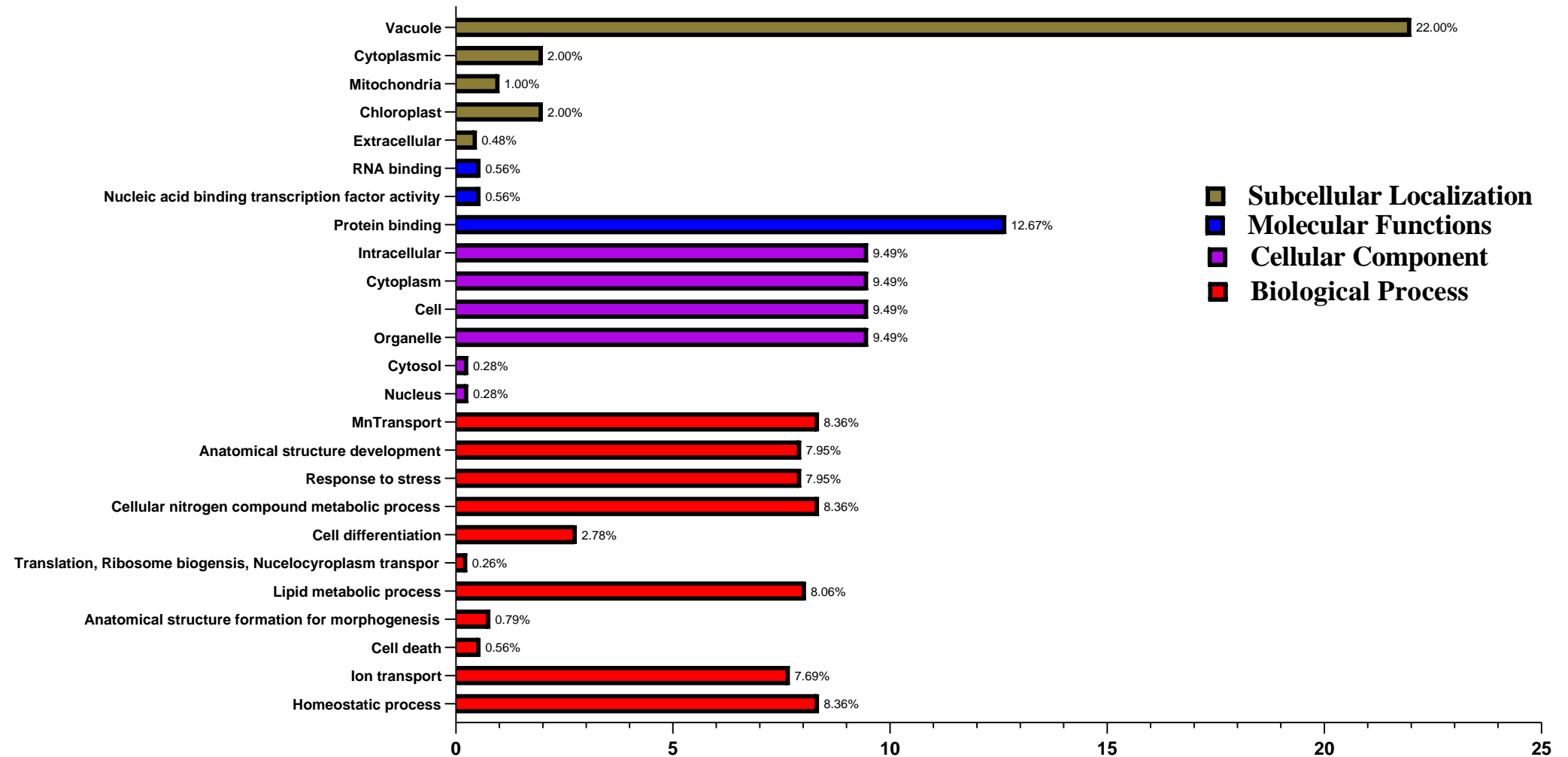

Supplement: Supplementary file 2 [file DataSheet_2.pdf]
